# Inmembrane, a bioinformatic workflow for annotation of bacterial cell-surface proteomes

**DOI:** 10.1186/1751-0473-8-9

**Published:** 2013-03-19

**Authors:** Andrew J Perry, Bosco K Ho

**Affiliations:** 1 Department of Biochemistry, Monash University, Melbourne, Australia; 2 Monash eResearch Centre, Monash University, Melbourne, Australia

**Keywords:** Bioinformatics, Proteomics, Membrane protein, Python, Bacterial

## Abstract

**Background:**

The annotation of surface exposed bacterial membrane proteins is an important step in interpretation and validation of proteomic experiments. In particular, proteins detected by cell surface protease shaving experiments can indicate exposed regions of membrane proteins that may contain antigenic determinants or constitute vaccine targets in pathogenic bacteria.

**Results:**

Inmembrane is a tool to predict the membrane proteins with surface-exposed regions of polypeptide in sets of bacterial protein sequences. We have re-implemented a protocol for Gram-positive bacterial proteomes, and developed a new protocol for Gram-negative bacteria, which interface with multiple predictors of subcellular localization and membrane protein topology. Through the use of a modern scripting language, inmembrane provides an accessible code-base and extensible architecture that is amenable to modification for related sequence annotation tasks.

**Conclusions:**

Inmembrane easily integrates predictions from both local binaries and web-based queries to help gain an overview of likely surface exposed protein in a bacterial proteome. The program is hosted on the Github repository http://github.com/boscoh/inmembrane.

## Background

**Figure 1:**
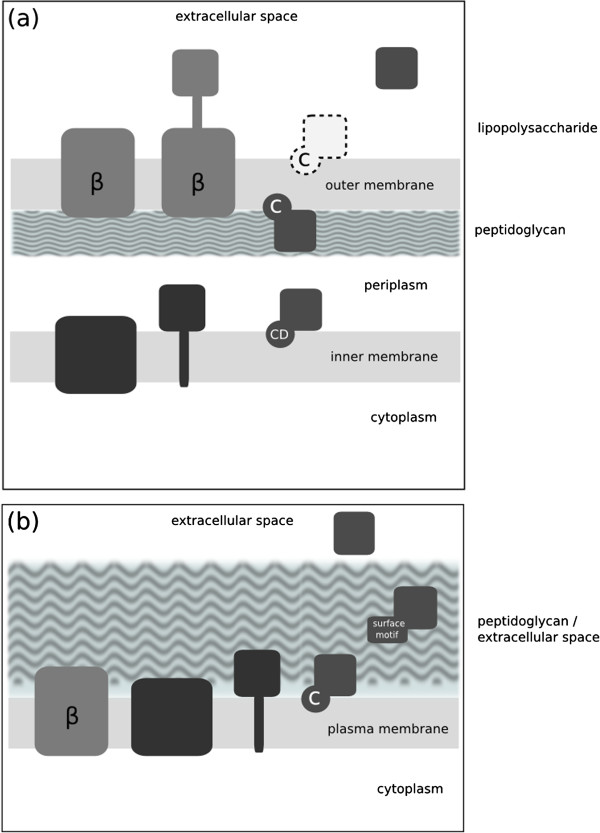
**Topologies represented in Gram-negative bacterial inner membrane include (left to right) polytopic transmembrane proteins, monotopic transmembrane proteins and lipoproteins on the periplasmic side of the membrane which are anchored via a lipid moeity covalently attached to the N-terminal cysteine ("CD", where "D" denotes an Asp outer membrane avoidance signal at position 2 (Masuda et al. 2002)).** The outer membrane also contains lipoproteins, usually on the inner leaflet exposed to the periplasm, however unlike the inner membrane the outer membrane contains ß-barrel membrane proteins ("beta"), some with large extracellular domains exposed on the surface. Akin to the Gram-negative inner membrane, the Gram-positive inner membrane contains mono and polytopic transmembrane proteins and lipoproteins. Gram-positive bacteria also display surface proteins associated covalently or non-covalently with the cell wall peptidoglycan layer via a number of "surface motifs", such as the LPxTG, LysM. Some proteins are also secreted into the extracellular milieu. A subset of Gram-positive bacteria (the Acinetobacterace) have also been shown to contain ß-barrel membrane proteins in their plasma membrane.

A common task in bioinformatics is to integrate the results of protein prediction programs to deduce complex properties of proteins. In studies of membrane proteomes, quick annotation of an experimentally detected set of the proteins can help highlight sequences of unexpected localization, and can alert researchers to possible contamination from other subcellular fractions. Ultimately, a concise summary of the properties of the detected membrane proteins in a particular proteomic dataset allows meaningful comparisons between different bacterial strains, species, and their responses in membrane remodeling to host and environmental challenges (Figure 1).

A number of published software packages exist for global prediction of subcellular localization of bacterial proteins. Most notable is PSORTb v3.0 [1] which predicts general subcellular localization for Gram-positive, Gram-negative and Archaeal protein sequences. CELLO [2] is a web accessible Support Vector Machine-based classifier that predicts localization of Gram-positive, Gram-negative and eukaryotic proteins. Some predictors and databases have been developed with a focus solely on Gram-positive surface proteins. Both Augur [3] and LocateP [4] are pipelines wrapping existing specific localization predictors, and provide web accessible databases of pre-calculated subcellular localization for Gram-positive proteomes. While the source code for PSORTb 3.0 is available under an open source license, the code for the other annotation pipelines discussed is not generally available for download.

An extension to general membrane localization prediction is the analysis of membrane protein topology to identify prominent surface exposed loops. These potentially surface exposed (PSE) proteins are of particular interest since they constitute attractive vaccine candidates. One existing workflow for annotation of PSE proteins is the program SurfG+ [5], which focuses on Gram-positive bacterial proteomes. SurfG+ is a Java program that carries out batch processing of several standard bioinformatic tools to specifically predict proteins that protrude out of the peptidoglycan layer of the bacterium. These predictions are intended to identify a set of proteins that would be accessible in cell-surface protease shaving experiments. SurfG+ itself does not carry out any computationally intensive analysis, but rather leverages the results of a transmembrane helix predictor (TMMOD) [6], a secretion signal predictor (SignalP) [7], a lipoprotein signal predictior (LipoP) [8] and a sequence alignment for protein profiles (HMMER) (http://hmmer.org).

Nevertheless, SurfG+ suffers several problems that plague much bioinformatic software. Despite being published in 2009, the URL mentioned in the original reference no longer exists. We were able to find a source-code repository (https://mulcyber.toulouse.inra.fr/projects/surfgplus) but we were not able to get the program to work, due in part to dependencies that are not longer generally available for download.

Since the core algorithm in SurfG+ is relatively straightforward, we decided to replicate and expand upon the functionality of SurfG+ by writing inmembrane in a modern scripting language. This lead to considerable simplification and clarification of the code base. Compared with the SurfG+, which is has 5,731 lines of source code (SLOC) (SVN revision 48, SLOCCount v2.26) primarily in Java, inmembrane, without dependencies, is around ~2400 SLOC of Python code and includes additional functionality not offered by SurfG+. The smaller code base is substantially easier to reuse and repurpose for other users. Here, we discuss the issues involved in writing robust and accessible bioinformatic source code.

## Methods and implementation

**Figure 2:**
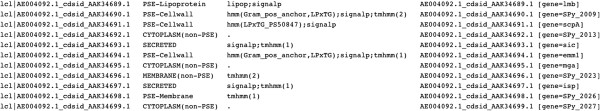
An example of inmembrane output using the gram_pos protocol.

inmembrane is primarily designed to be run locally via the command line. The input is a set of sequences in FASTA format, the output is plain text (Figure 2), including a summary table as well as an output file in comma-separated-value (CSV) format suitable for import into spreadsheet software or scripted text processing.

A set of unit tests, executable via the commandline option “inmembrane_scan --test” enables users and developers to quickly verify if their inmembrane installation, with dependencies, is functioning as expected.

### Gram-positive protocol

The inmembrane Gram-positive surface protocol leverages a number of existing single localization predictors, including transmembrane topology prediction, to deduce the likely subcellular localization and expected surface exposure of each protein in a given proteome. Each sequence is annotated by every predictor, and these annotations are used by the business logic of inmembrane to classify proteins as potentially surface exposed ("PSE"), "Secreted", or the non-exposed classes "Cytoplasmic" and "Membrane".

Annotations applied are as follows. HMMER 3.0 [9] searches using hidden Markov models (HMM) derived from Pfam and Superfam are used to detect known Gram-positive surface sequence motifs. These include LPxTG [10] [PF00746 and the HMM used by SurfG+ [5], GW repeat domains [11] [Superfam models 0040855, 0040856, 0040857], peptidoglycan (PG) binding domain (Type 1) [12] [PF01471, PF08823, PF09374]], Choline binding repeats [13], [PF01473] LysM domain [14] PF01476, Cell-wall binding domain (Type 2) [15], [PF04122], S-layer homology domain [16] [PF04122] motifs and the NLPC_P60 cell wall associated domain [17] [PF00877]. PFAM HMMs are from most recent version of at the time of writing, release 26.0.

Lipoprotein signals are detected using LipoP [8], and signal sequences are detected using SignalP [7], including detection of signal peptidase cleavage sites.

The presence and topology of transmembrane segments in helical membrane proteins is predicted using TMHMM v2.0 [18] and/or MEMSAT3 [19]. Since MEMSAT3 executes a PSI-BLAST search to gather homologous sequences, it is considerably slower than TMHMM, and as such, is turned off by default.

**Figure 3:**
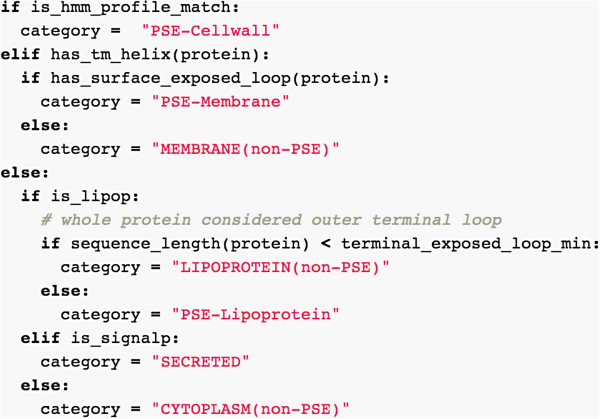
**Main logic classifying subcellular localization and potential surface exposure for Gram-positive protein sequences, expressed in Python code.** This algorithm was adapted from SurfG+. The function has_surface_exposed_loop evaluates whether the extracellular loops are sufficiently long to be exposed out of the peptidoglycan layer. The rule adapted from SurfG+is a minimum length of 50 amino acids for terminal loops, and 100 amino acids for internal loops.

Inmembrane collates the results of each analysis, and using the predicted topology of the intergral membrane proteins detected, predicts potentially surface-exposed loops following the algorithm used by SurfG+ (Figure 3). By default, external terminal regions longer than 50 residues and external loops longer than 100 residues are considered to be potentially surface exposed. These values were previously experimentally derived based on membrane shaving experiments with S. pyrogenes and may need modification to suit other species with different cell wall thickness [5].

### Tests with Gram-positive bacteria

The field of bioinformatics changes quickly, and in the few years since the release of SurfG+, some of its dependencies are no longer readily available. For instance TMMOD is no longer released as a binary and SignalP has progressed to Version 4.0. As a result we could not use the same version of the binaries used in SurfG+. Nevertheless, inmembrane produces comparable results to SurfG+ for the 5 bacterial genomes originally tested (Table 1). This can also be compared to PSORTb 3.0 classification for the same organisms (Table 2).

**Table 1:** Comparison of inmembrane Gram-positive protocol results with SurfG+

	**S. pyogenes**		**L. acidophilus**		**L. johnsonii**		**L. gasseri**		**L. bulgaricus**	
Accession	EMBL:AE004092		EMBL:CP000033		EMBL:AE017198		EMBL:CP000413		EMBL:CR954253	
Program	S	i	S	i	S	i	S	i	S	i
CYTOPLASM(non-PSE)	1243	1234	1290	1280	1248	1234	1262	1240	1132	1119
MEMBRANE(non-PSE)	236	238	315	329	357	355	298	302	244	261
PSE(total)	140	172	169	189	176	203	157	188	116	137
SECRETED	78	52	88	64	40	29	38	25	70	45
Total	1697	1696	1862	1862	1821	1821	1755	1755	1562	1562

Columns labelled 'S' are SurfG+ results and 'i' are inmembrane results. Some inmembrane subclasses have been combined to directly compare with SurfG+ (i.e. PSE(total) = PSE-Membrane + PSE-Cellwall + PSE-Lipoprotein).

**Table 2:** PSORTb 3.0 classifications for Gram-positive genomes

	**S. pyogenes**	**L. acidophilus**	**L. johnsonii**	**L. gasseri**	**L. bulgaricus**
Accession	EMBL:AE004092	EMBL:CP000033	EMBL:AE017198	EMBL:CP000413	EMBL:CR954253
Cellwall	24	46	26	27	19
Cytoplasmic	884	855	826	804	743
Cytopl. Membrane	432	519	548	489	440
Extracellular	28	32	16	13	15
Unknown	323	402	394	419	307
Unknown/multiple	5	8	11	3	5
Total	1696	1862	1821	1755	1529

PSORTb 3.0 analysis of the genomes in Table 1 analysis, derived from PSORTdb. Direct comparison of classifications is difficult since PSORTb, SurfG+ and inmembrane each annotate with different classes (for instance, the basic PSORTb classification does not differentiate between lipoproteins and cytoplasmic membrane proteins, and SurfG+ and inmembrane do not include an “Unknown”, but instead classify sequences without any detected features as cytoplasmic by default).

### Gram-negative protocol

In addition to the Gram-positive surface protocol, we have also implemented a protocol for summarizing subcellular localization and topology predictions for Gram-negative bacterial proteomes. Gram-negative bacteria have both a cytoplasmic (inner) membrane, a periplasmic space, a peptidoglycan layer and an outer membrane decorated in lipopolysaccharide (Figure 1). Membrane proteins integral to the inner membrane contain hydrophobic helical transmembrane segments, analogous to the Gram-positive cytoplasmic membrane, while the proteins embedded in the outer membrane form ß-barrels composed of amphipathic ß-strands. Lipoproteins in Gram-negative bacteria can be associated with the inner or the outer membrane.

Potential signal sequences of the general (Sec) secretory pathway are predicted using SignalP. Twin-Arginine translocase (Tat) signals are predicted using TatFind [20] and a profile HMM built from the Prosite [21] Tat sequence set (PS51318). Transmembrane helices and topologies of inner membrane proteins are predicted using TMHMM and optionally with MEMSAT3. As with the Gram-positive protocol, lipoproteins were predicted using LipoP, however the Gram-negative protocol additionally detects the “Asp+2” inner-membrane retention signal [22] to differentiate between lipoproteins transported to the outer membrane (LIPOPROTEIN(OM)) and those retained on the periplasmic side of the inner membrane (LIPOPROTEIN(IM)).

The topology of integral inner membrane proteins is analysed using the same ‘potentially surface exposed’ loops algorithm as the Gram-positive protocol, however in this case sequences are classified as 'IM', 'IM(cyto)', 'IM(peri)' and 'IM(cyto+peri)' to indicate proteins with long cytoplasmic and/or periplasmic loops or domains. Experimentally, large periplasmic domains may be accessible to protease shaving when the outer membrane has been disrupted, such as in spheroplasts generated using outer membrane permeabilization agents. Unlike the Gram-positive plasma membrane, the Gram-negative inner membrane is not decorated with LPS and as such periplasmic loops and domains of intergral membrane proteins are expected to be more easily accessed by protease once the outer membrane is permeabilized. We have chosen a length of 30 residues as a conservative threshold (the 'internal_exposed_loop_min' setting) for annotating cytoplasmic ('+cyto') and periplasmic ('+peri') loops or domains. This should be modified as required to suit the purpose of the user.

Outer membrane ß-barrel proteins are predicted using the BOMP [23], TMB-HUNT [24] and TMBETADISC-RBF [25] web services. By default, high scoring sequences that are more likely to be true-positives are annotated as 'OM(barrel)' and are not strictly required to have a predicted signal sequence (BOMP score >= 3 and TMBHUNT probability >= 0.95). Lower scoring sequences (1 < BOMP score >= 2 and 0.5 < TMBHUNT probability >= 0.94, and all TMBETADISC-RBF positive predictions) must contain a predicted signal sequence to be annotated as an outer membrane barrel. We have also implemented an interface to TMBETA-NET [26] which can be used to annotate the predicted number (and location) of membrane spanning strands for outer membrane ß-barrels, however this method is disabled by default since it is prone to false positives for multidomain proteins where both a membrane ß-barrel and an additional soluble domain are present [27].

Proteins containing a predicted N-terminal Sec or Tat signal sequence without internal transmembrane segments or a ß-barrel classification are annotated as 'PERIPLASMIC/SECRETED'. If no membrane localization or signal sequence is detected, the protein is annotated at 'CYTOPLASMIC'. Currently, the protocol does not explicitly detect localization for some secrected proteins without a signal sequence, such as those that contain Type 3 secretion signals or flagellar and pilus components.

### Future protocols

inmembrane is designed such that new workflows for annotation of membrane proteomes can be added easily. Wrappers for programs that annotate a sequence with a particular feature can be added to inmembrane/plugins/ following the example of existing plugins. The inmembrane/plugin/signalp4.py and inmembrane/plugin/lipop1.py plugins provide good templates for adoption and modification. In the simplest case, this means that if a superior method for signal peptide, transmembrane segment or lipoprotein prediction is developed, or an existing method becomes unavailable, it will be straightforward to write a new plugin wrapping it for inclusion in the protocol. New protocols can be added to the inmembrane/protocols directory, and selected for execution by changing protocol parameter in the inmembrane.config file. Currently, we have implemented two protocols, gram_pos, for prediction of PSE proteins in Gram-positive bacteria, and gram_neg, for general annotation of Gram-positive subcellular localization.

## Discussion

### Software distribution and long term availability

The problem of long term of durability of computational biology software is a significant issue for both downloadable packages and hosted web services [28].

Perhaps the single most important step in improving the quality and long term availability of code is to distribute it on a publicly available open-source repository. We believe that the use of a dedicated repository provides many advantages over the typical strategy of hosting software on an academic server. For inmembrane, we chose to host the source code on Github, which provides excellent code-browsing facility, code history, download links, and robust well-defined URL links. Github provides excellent usage statistics to measure the impact of the software, which obviates the need for the dreaded login and registration pages. Importantly, storing the software in a well-supported repository with a clear business model means the source code is likely to remain accessible in the long term, something that historically many academic labs have shown they cannot provide (Veretnik et al., 2008). If you were to come across an abandoned project on Github, it would be trivial to 'fork' the project, producing your own duplicated copy of the code which can be changed and improved. To this end, we have applied a liberal BSD license to inmembrane to enable the widest possible reuse.

While we have taken strategies to ensure inmembrane itself is likely to remain available in the long term, we cannot control the availability of many of it's downstream dependencies, which are either web services or binaries which cannot be freely redistributed. A key design decision in inmembrane is the use of loosely-coupled plugins for each external program or web service. This allows developers to easily ‘route around the damage’ if a particular web service or piece of software becomes unavailable by replacing one sequence analysis package with an alternative that gives similar (if not identical) annotations. In the long term, we hope that any proprietary components can be replaced with more durable open source dependencies as they may become available.

### Program setup and workflow

The heart of inmembrane is simple: it takes FASTA sequences, sequentially provides them as input to a number of external sequence analysis programs, processes their output and provides the combined annotations as plain text output. The bulk of the computation applied by inmembrane itself lies in the parsing of the text output of the external programs and the post-processing business logic.

As inmembrane integrates the output of a large number of external dependencies, there are many potential points of failure. As such, inmembrane saves all intermediate output into a results folder, and a comprehensive set of unit tests is provided to help diagnose issues with dependencies. If the user requires all local external binaries, then inmembrane is restricted to a Linux platform. However, if web-based modules are selected, then the only external local dependency is HMMER, which allows inmembrane to run on any Unix-like system.

It is not uncommon for scientific software packages to disperse configuration information throughout the header regions of multiple scripts and/or shell environment variables, and users are asked to search through the program and modify the source code. While convenient for the original programmer, this can be frustrating and confusing even for expert users. A far better model is to isolate the configuration concerns to one clear place with sensible defaults. Following this model, inmembrane reads configuration information from an explicit configuration file inmembrane.config, where a default version is auto-generated if it is not initially found.

Since the configuration file for inmembrane is itself a Python dictionary, expert users can write a short Python script that incorporates a specific configuration dictionary and execute inmembrane directly. This provides a convenient record of each individual analysis, as well as a file that can be executed through a file-manager by double-clicking (an example is provided in the script inmembrane_example.py).

### Scripting languages

The virtues of Python as a language for solving problems in life science research have been previously recognized [29]. One potential downside of Python is it's slower execution speed for computationally intensive tasks when compared with compiled languages, or just-in-time compiled languages such as Java. Since inmembrane delegates most of the computationally intensive tasks to external programs, the wrapping, text parsing and analysis code in Python does not become a bottleneck in the overall processing speed.

Programs written in Java almost always follow an object-oriented programming (OOP) approach. Although OOP provides advantages when architecting large enterprise systems, it's overuse for small projects can be a disadvantage. In the recommended Enterprise Java style of programming used in SurfG+, objects are created through several layers of abstract classes where each field in an object needs to be explicitly specified. To change a field in a data structure, there are at least 6 places in 3 different files where the code that needs to be changed, which severely restricts the ease of modification for those unfamiliar with the code base. Whilst this level of hierarchy is useful in programs that have highly interdependent data-structures, this is not the case here and adds otherwise unneeded levels of complexity.

Using a modern scripting language such as Python results in cleaner code, where the advantages arise mostly from the use of standard dynamic language features, which otherwise would require the creation of complex object hierarchies in Java. Another advantage is portability, where the Python source code itself is directly executable. This allows a faster development cycle when modifying the source code compared with the edit-compile-run cycle required for compiled languages.

### Simple data structures facilitate simple text parsing

In inmembrane, the standard Python dictionary is used to provide a flexible way to represent data and allow extremely simple parsing code to be written. The Python ‘dictionary’, which is conceptually similar to a ‘hash table’ or ‘hash map’ in other languages, consists of a set of key-value pairs, where keys and values can be any type of data structure - strings, integers, floats, or even other dictionaries.

The core data structure used by inmembrane is a flat Python dictionary called proteins, indexed by sequence identifiers. Let’s say our FASTA file contains the Streptococcus pyogenes C5a peptidase sequence with the ID 'C5AP_STRPY'. The properties of C5AP_STRPY would then be found in proteins['C5AP_STRPY'], which is itself a dictionary. proteins['C5AP_STRPY'] contains any arbitrary number of different properties, also accessed as key-value pairs. For instance, the sequence length of the 'C5AP_STRPY' sequence would be stored in proteins['C5AP_STRPY']['sequence_length']. This data structure can capture the results of most basic sequence analyses, where new properties are added to proteins on the fly. The use of a dynamic flat dictionary avoids much of the boilerplate code involved with an OOP style programming.

**Figure 4:**
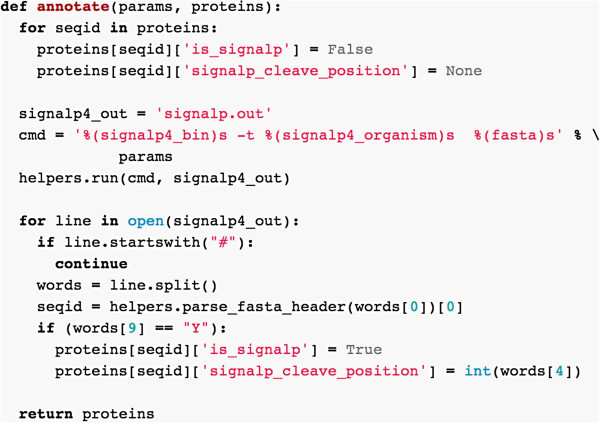
**Example of parsing code in the signalp4 plugin.** The entire function responsible for processing SignalP output. helpers is an inmembrane module with utility functions.

If we use a dictionary to represent our data structure, then the main work in inmembrane of running other programs and processing their text output can be encapsulated into a simple function. For example with SignalP, we define a function signalp.annotate(params, protein) which takes the main protein data structure as input. The function runs the external SignalP binary, and then parses the text output. Text processing is very easy to write in Python and the extracting the minimum information required by our protocol from SignalP output can be achieved with around 15 lines of code (Figure 4).

As signalp.annotate cycles through the text output of SignalP, for each protein, if a secretion signal is found, a new property is added: proteins['C5AP_STRPY']['is_signalp'] = True. We can thus abstract the main program loop as running a series of functions of the generic form program.annotate(params, protein). This simple plugin API allows inmembrane to be extended with new analysis modules that annotate the proteins dictionary.

Inmembrane avoids hard coding references to external data files where appropriate. For example, for HMMER peptide motif matching, instead of hard-coding the sequence profiles to search (as in SurfG+), inmembrane dynamically searches the directory defined in the protocol (e.g. protocols/gram_pos_profiles) for sequence profiles, which are used for HMMER analysis. New profiles can be processed by simply dropping them into this directory.

### Interfacing with web services

The simple plugin framework used by inmembrane can be used to interface with remote web services as well as locally installed software. Many useful bioinformatics sequence analysis tools are provided with an HTML form based front end designed for web browsers, but with no official machine readable web API, and no downloadable standalone version of the software. While researchers may neglect to provide these interfaces for a multitude of reasons, for end-users the lack of a standalone version or a web API makes automated use for large scale analyses, such as that carried out by inmembrane, somewhat awkward and inconvenient. Several of the published tools for the detection of outer membrane ß-barrel proteins we wished to use as part of the inmembrane 'gram_neg' protocol only provide a browser based interface, and some only allow submission of a single protein sequence at one time. To solve this problem we chose to implement automated queries to these web interfaces using the twill library [30], with subsequent parsing of any HTML output using the BeautifulSoup library [31].

**Figure 5:**
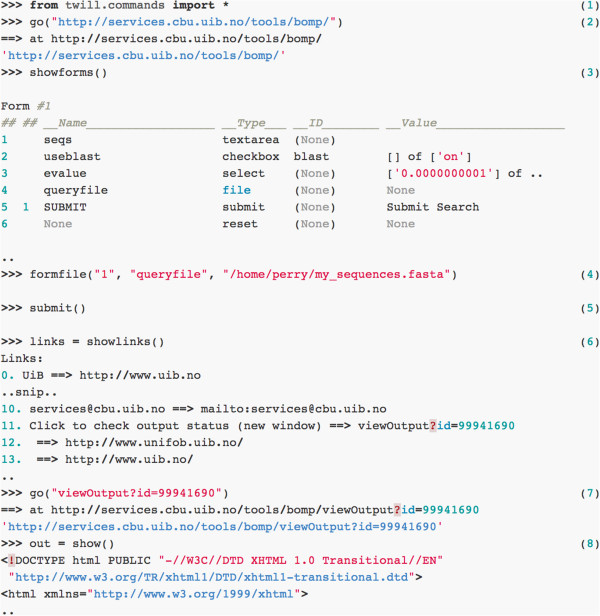
**An example of interfacing with the BOMP ß-barrel outer membrane protein predictor (Berven et al., 2004) web site using twill on the Python interactive commandline.** twill essentially behaves like a headless web-browser. Lines with >>> denote inputs to the Python interactive command line, while other lines are output from twill (1) First the appropriate commands from the twill library are imported. (2) We navigate to the BOMP website, which silently downloads the HTML page and (3) show a summary of the forms on that page, including field names and input types. (4) We then use the formfile function to associate a local file with the queryfile FILE input field. Calling submit() (5) is equivalent to clicking the SUBMIT button defined in the form. After a short delay, an intermediate page is returned, and we can list the hyperlinks on this page using (6) showlinks(), and assign them to a variable (links, a Python list). We can then navigate to the appropriate result page (7) and assign the HTML text of this page to a variable (out) (8) for downstream parsing using BeautifulSoup. This type of interactive exploration can be easily expanded into an inmembrane plugin to programmically interface with the web service.

When writing a wrapper for a new service, commands to interface with a web form can be easily tested directly on the Python command-line, or by using twill itself in interactive mode (Figure 5). This allows for quick prototyping of new web scrapers, prior to implementation as an inmembrane plugin.

In it's simplest form, a web service API is essentially an agreement between a service provider and their end-users on a machine readable, predictable and stable interface. Since 'screen scraping' as a method of interfacing with a sequence analysis tool does not use a well defined API with an implicit guarantee of stability, it can be prone to breakage when the format of the job submission or results page is changed even slightly. While we believe that the approach taken by twill and the robust parsing provided by BeautifulSoup will prevent many upstream changes breaking these wrappers, inevitably breakage will occur. In this case, the simplicity and ease of modifiability of the code base becomes a key feature that allows expert users to fix plugins if and when it is required.

The use of web services constitutes a usability trade-off against the use of local external binaries. Using web services significantly simplifies the installation procedure for users of inmembrane, however correct operation requires that the Internet is readily available, that the service provider has ensured good up-time, and that the web-service protocol will not be silently changed or deprecated. Some services also impose daily usage limits that may prevent very large scale analysis. While many popular services are maintained centrally by large organizations to ensure their stability, smaller services are often maintained by a single graduate student, placing significant risk on their long term availability. In contrast to the convenience of web services, installing local binaries can be time consuming. Each external binary has to be installed and tested, often requiring reasonable aptitude with the Unix command line. Additionally, binaries may not be cross-platform: for instance, the full set of external binaries required by inmembrane is only available for Linux. Nevertheless, the advantage of locally installing dependencies is that once installed, the user can be confident in the future operation of inmembrane.

## Conclusions

inmembrane provides a clean bioinformatic pipeline to analyze proteomes for membrane proteins that contain exposed regions outside of the membrane. Testing has shown that the results derived from the inmembrane Gram-positive protocol are comparable to previously published analysis. The inmembrane software has been written in a style of programming intended to enhance readability and extensibility of the code, and we sincerely hope that inmembrane will be modified and improved by other researchers. We welcome other researchers to join us on Github. modified and improved by other researchers. We welcome other researchers to join us on Github.

### Availability and requirements

**Project name:** inmembrane

**Project home page:**http://boscoh.github.com/inmembrane

**Operating systems:** Linux

**Programming language:** Python

**Other requirements:** HMMER, SignalP, LipoP, TMHMM or MEMSAT3. An Internet connection is required for web services such as BOMP, TMB-HUNT and TMBETADISC-RBF.

**Licence:** BSD Licence (2-clause)

Any restrictions to use by non-academics: Use of inmembrane itself is unrestricted, however many of the dependencies require special licensing for non-academic use.

## Abbreviations

PSE: Potentially surface exposed; OMP: Outer membrane protein; HTML: Hypertext Markup Language; API: Application programming interface; OOP: Object-oriented programming; BSD: Berkley Software Distribution; LPS: Lipopolysaccharide; CSV: Comma-separated-value; SLOC: Source lines of code; URL: Uniform resource locator

## Competing interests

The authors declare that they have no competing interests.

## Authors' contributions

Both AJP and BKH wrote and drafted the manuscript, and coded and tested the software described. All authors read and approved the final mansucript.
